# Influence of Substitutional Defects in ZIF-8 Membranes on Reverse Osmosis Desalination: A Molecular Dynamics Study

**DOI:** 10.3390/molecules26113392

**Published:** 2021-06-03

**Authors:** Terence Zhi Xiang Hong, Liming You, Madhavi Dahanayaka, Adrian Wing-Keung Law, Kun Zhou

**Affiliations:** 1 Environmental Process Modeling Centre, Nanyang Environment and Water Research Institute, 1 Cleantech Loop, Singapore 637141, Singapore; TERE0016@e.ntu.edu.sg (T.Z.X.H.); daha0001@e.ntu.edu.sg (M.D.); cwklaw@ntu.edu.sg (A.W.-K.L.); 2 Interdisciplinary Graduate School, Nanyang Technological University, 50 Nanyang Avenue, Singapore 639798, Singapore; liming001@e.ntu.edu.sg; 3 School of Mechanical and Aerospace Engineering, Nanyang Technological University, 50 Nanyang Avenue, Singapore 639798, Singapore; 4 School of Civil and Environmental Engineering, Nanyang Technological University, 50 Nanyang Avenue, Singapore 639798, Singapore

**Keywords:** molecular dynamics simulation, reverse osmosis, seawater desalination, zeolitic imidazolate frameworks

## Abstract

In this study, molecular dynamics simulation is used to investigate the effects of water-based substitutional defects in zeolitic imidazolate frameworks (ZIF)-8 membranes on their reverse osmosis (RO) desalination performance. ZIF-8 unit cells containing up to three defect sites are used to construct the membranes. These substitutional defects can either be Zn defects or linker defects. The RO desalination performance of the membranes is assessed in terms of the water flux and ion rejection rate. The effects of defects on the interactions between the ZIF-8 membranes and NaCl are investigated and explained with respect to the radial distribution function (RDF) and ion density distribution. The results show that ion adsorption on the membranes occurs at either the nitrogen atoms or the defect sites. Complete NaCl rejection can be achieved by introducing defects to change the size of the pores. It has also been discovered that the presence of linker defects increases membrane hydrophilicity. Overall, molecular dynamics simulations have been used in this study to show that water-based substitutional defects in a ZIF-8 structure reduce the water flux and influence its hydrophilicity and ion adsorption performance, which is useful in predicting the type and number of defect sites per unit cell required for RO applications. Of the seven ZIF-8 structures tested, pristine ZIF-8 exhibits the best RO desalination performance.

## 1. Introduction

Zeolitic imidazolate frameworks (ZIFs) are a subset of metal-organic frameworks (MOFs) that are topologically isomorphic with zeolites due to their metal-imidazole-metal angles resembling the 145° Si-O-Si angle in zeolites. They consist imidazolate linkers that are linked to tetrahedrally-coordinated transition metal ions via dative bonds [1]. While different types of ZIFs exist (e.g., ZIF-69, ZIF-70, ZIF-72, ZIF-80, and ZIF-82), their individual topologies and chemical compositions correspond to different structures with varying pore sizes, adsorption capacities, and separation performance [2,3,4]. One type of ZIF commonly applied in both gas and water separation is ZIF-8, in which N atoms in the imidazolate linkers exhibit dative bonds with the Zn atoms. Due to the hydrophobic pores of ZIF-8 as a result of stable tetrahedral clusters formed between the Zn atoms and the inhibitive structure of the imidazolate linkers, ZIF-8 is highly resistant to water degradation [5]. This renders ZIF-8 a viable candidate for desalination applications such as reverse osmosis (RO), which is commonly implemented for seawater treatment [5,6].

In RO seawater desalination, ZIF-8 is mainly employed as an additive for improving the desalination performance of thin-film-composite RO polymer membranes by increasing the water flux through them while maintaining a high NaCl rejection rate. Such an increase can be attributed to additional channels as a result of the induced spacings between the polymer matrix and the ZIF-8 interfaces, the high porosity of ZIF-8 that allows more water molecules to permeate the membrane in a given time, and the hydrophobicity of the ZIF-8 pores, in which the weak affinity between the water molecules and the pores allows water to pass through the membrane rapidly. A high ion rejection rate is maintained when ion adsorption occurs on the surface of the membrane, thereby facilitating atomic interactions between atoms in the ZIF-8 structure and ions in the water.

Duan et al. [7] investigated the usage of ZIF-8 as nanofillers in thin-film-nanocomposite (TFN) membranes for RO applications. The results indicate that the rate of water transport will be higher if ZIF-8 (0.47 L µm/m^2^·h·bar) is used instead of zeolite (0.06 L·µm/m^2^·h·bar) [7]. ZIF-8 exhibits superior compatibility with a polyamide (PA) matrix, which allows for the removal of nonselective spacings between ZIF-8/PA interfaces that can potentially lower the NaCl rejection rate [6]. Furthermore, due to the increasing roughness of the TFN membrane with ZIF-8 loading, its surface will become more hydrophilic, which reduces fouling. Indeed, the water permeance of a TFN membrane loaded with ZIF-8 is 162% higher than that of a pristine PA membrane [6].

In an molecular dynamics (MD) simulation study on ZIF-8 RO membranes, Hu et al. explored a pristine ZIF-8 membrane whose pores were orientated in a direction parallel to the general water flow direction to allow for maximum water flow through the membrane [5]. Their results indicate a NaCl rejection rate of 100% [5]. Although a number of relatively small Na^+^ ions could enter the membrane through the larger cavities, the sieving effect of the ZIF-8 apertures and the rigid and defect-free nature of the ZIF-8 membrane limited the movement of these ions within it [5]. By keeping the applied pressure constant while varying the temperature of the system, the activation energy of water permeation for the membrane was estimated to be 24.4 kJ/mol according to the Arrhenius equation [5], which is comparable to those of aromatic polyamide composite membranes [8]. However, the study did not account for defects within the ZIF-8 framework that can potentially influence its RO desalination performance.

While ZIF-8 is highly resistant to hydrolysis, its Zn–N dative bonds may still break eventually and create defects. Based on the study by Zhang et al. [9], ZIF-8 crystals were observed to degrade in water and release Zn^2+^ ions with a low ZIF-8 crystal to water mass ratio that increases the rate and extent of ZIF-8 dissolution [9]. Due to the disappearance of the ZIF-8 layer, a sharp increase in the water pervaporation flux occurred [9]. In another study on the static stability of ZIF-8 in pure water, the ZIF-8 crystalline structure was determined to be almost completely ruined after being submerged in water for 72 h [9].

In a computational characterization study of water-induced point defects in ZIF-8 by Zhang et al., the formation of linker and Zn substitutional defects in ZIF-8 were discovered to be thermodynamically favorable, as such defective ZIF-8 structures exhibit lower energy values than pristine ZIF-8, with the formation of linker defects occurring at a higher probability. However, since the strong Zn–N dative bonds must be broken to form such defects [10,11], the formation of large concentrations of defects at room temperature is highly unlikely. By studying the diffusion of various molecules through ZIF-8 membranes with linker defects, Han et al. established that low concentrations of linker defects can possibly increase the local hopping rate for molecular diffusion [12]. Therefore, the presence of a small number of substitutional defects may impact the RO desalination performance of a ZIF-8 membrane [10].

In this study, MD simulations were conducted to investigate the effects of water-induced substitutional defects in ZIF-8 membranes on their RO desalination performance. Seven ZIF-8 membranes composed of pristine ZIF-8, three ZIF-8 structures with 1, 2, and 3 Zn substitutional defects in their respective unit cells, and three ZIF-8 structures with 1, 2, and 3 linker substitutional defects in their respective unit cells. Their desalination performance was evaluated in terms of the water flux and ion rejection rate during the initial period of the RO process before they were saturated with ions. The effects of pressure and temperature on water transport within these membranes were also investigated.

## 2. Simulation Details

To create defective ZIF-8 structures, the Zn atoms and linker groups in a perfect cubic ZIF-8 unit cell were replaced with two types of water-induced point defects to varying degrees [10]. Based on the study by Zhang et al. [10], each Zn atom defect was created by removing a Zn atom and replacing it with two H atoms, which were randomly bonded to two N atoms that were originally bonded to the missing Zn atom (Figure S1a). To create each linker defect, the organic linkers were replaced by an OH-H-OH functional group whose O atoms were bonded to the Zn atoms (Figure S1b). As mentioned previously, three Zn-defective unit cells (Zn1, Zn2, and Zn3) were created in this study, in which one, two, and three Zn atom defects were present in each respective unit cell, and three linker-defective unit cells (linker1, linker 2, and linker3) were created, in which one, two, and three linker defects were present in each respective unit cell. Since it was highly challenging to estimate the absolute equilibrium defect concentration pertaining to each ZIF-8 unit cell [10,12], this theoretical study was conducted under the assumption that the defective ZIF-8 membranes will remain chemically stable throughout the entire simulation with up to three defects in each of their respective unit cells. A defect-free ZIF-8 unit cell was selected as a point of reference to assess the RO desalination performance of the membranes corresponding to the six defective ZIF-8 unit cells.

To account for the varying concentrations and types of defects within the respective defective ZIF-8 unit cells, geometry optimization was performed on the seven ZIF-8 unit cells, for which the optimized structures correspond to minimal ground state energies. In this study, the most stable ground-state configuration of each ZIF-8 structure was obtained through an iterative halving method in conjunction with comprehensive density functional theory calculations, which were conducted via the Vienna Ab initio Simulation Package (VASP) [13,14]. The Projector Augmented-Wave method [15] was selected for the pseudopotential formalism, and the Perdew−Burke−Ernzerhof (PBE) [16] parametrized functional with vdW-DF corrections was applied [17,18,19]. The calculations converged with a cutoff energy of 500 eV and a 1 × 1 × 1 Monkhorst−Pack k-mesh grid. 

**Figure 1:**
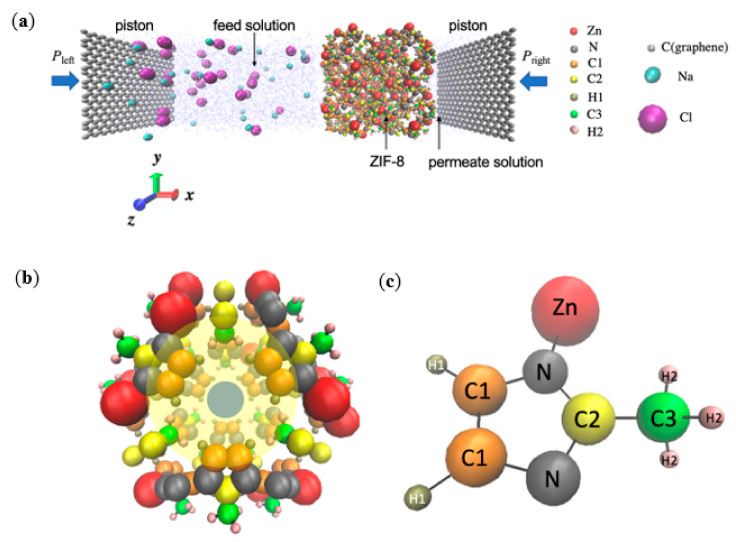
(**a**) Schematic of the simulation system for the pristine ZIF-8 membrane. The feed solution (Na^+^, Cl^−^, and water) and the permeate reservoir (pure water) are separated by a ZIF-8 membrane. Two graphene pistons at the two ends of the chambers are subjected to hydraulic pressures P_left_ and P_right_, respectively. (**b**) A representative unit cell of the ZIF-8 framework. The blue circle highlights an aperture, and the yellow circle highlights a cage. (**c**) The 2-methylimidazole structure exhibited by ZIF-8, in which the atoms are represented by the following color codes: Zn: red, N: grey, C1: orange, C2: yellow, H1: tan, C3: green, H2: pink, H (water) and O (water): blue, Na^+^: cyan, Cl^−^: purple, and C (graphene): silver.

Similarly to the set-up by Gupta et al. [20] and Hu et al. [5] for simulating an actual RO experiment, the simulation system consisted a ZIF-8 layer, which acts as the separation layer in ZIF-8 based RO composite membranes and is located between the feed and permeate solutions (Figure 1). The membrane and water bodies were further enclosed by two inert graphene pistons. The feed solution on the left contained 28 Na^+^ ions, 28 Cl^−^ ions, and 2453 water molecules, while the permeate solution on the right contained 593 water molecules. For the respective RO membranes, the number of atoms within each ZIF-8 membranes was as follows: pristine: 2208, Zn1: 2216, Zn2: 2224, Zn3: 2232, linker1: 2160, linker2: 2112, and linker3: 2064. The NaCl concentration of the feed solution was 0.6 M, which represents the typical NaCl concentration of seawater [21]. The dimensions of the feed solution were 70 × 34 × 34 Å^3^ and those of the permeate solution were 17 × 34 × 34 Å^3^. The ZIF-8 layer acting as the separation layer was positioned along the *yz*-plane and was composed of 2 × 2 × 2 ZIF unit cells. The two graphene pistons were parallel to the *yz*-plane, and each of them possessed dimensions of 3.4 × 34 × 34 Å^3^. Periodic boundary conditions were imposed along the *x-*, *y-*, and *z*-directions to ensure continuous feeding. Similarly to other studies that implemented periodic boundary conditions in all three directions [22,23,24], an additional space with a length of 73 Å was added along the *x*-axis to prevent the system from interacting with its images with respect to the *x*-direction. Overall, the dimensions of the entire simulation box were 200 × 35 × 35 Å^3^.

All MD simulations were based on the large-scale atomic/molecular massively parallel simulator (LAMMPS) package [25]. To model the water molecules, the commonly utilized and less complex TIP3P model was employed by setting the partial charges of O and H to −0.830e and 0.415e, respectively [26,27]. Such a model of water has also been implemented in other RO simulation studies pertaining to ZIF-8 [5] and ZIF structures with different linker functional groups [20]. Other atomic interactions were modeled by the Chemistry at HARvard Macromolecular Mechanics (CHARMM) force field [28], for which both the Lennard-Jones and coulombic terms possess an inner cut-off distance of 10 Å and an outer cut-off distance of 12 Å. The long-range Coulombic interactions were computed by the particle–particle–particle–mesh (PPPM) method with an accuracy value of 10^−6^ [29]. The partial charges and LJ parameters of the Na^+^ ions, Cl^−^ ions, and C atoms were adopted from previous studies [22]. Meanwhile, the partial charges and LJ parameters of the ZIF atom species were obtained from the classical Assisted model building with an energy refinement force field [30,31].

The entire model employs the constant volume and temperature (NVT) ensemble. The temperature of the system was kept constant at 300 K. To model the ZIF-8 membranes, the Zn, N, C2, and the atoms (H3, H4, and O) constituting the ZIF-8 defects were tethered to their initial positions by an imaginary spring with a spring constant of 100 Kcal/mole/Å^2^, and only limited movement of these atoms was allowed [32]. In contrast, the other atoms in the ZIF membranes could move freely, thereby representing flexible ZIF-8 membranes. Such a measure prevented the ZIF layers from collapsing under pressure and from deviating from their initial positions. The SHAKE algorithm was implemented with respect to the angles and bonds of the water molecules to render them rigid. A massive pressure difference was set to simulate the rapid movement of water molecules and ions through the ZIF-8 membranes within a short timeframe. After the model had undergone a minimization process for 0.7 ns with a timestep of 0.01 to assume a steady-state, a net pressure difference, Δ*P*, of 30 MPa was then applied along the *x*-direction, in which the pressure values of 30.1 MPa and −0.1 MPa were applied to the atoms constituting the graphene pistons in the feed chamber and the permeate chamber, respectively. Additional simulations were conducted under different pressure values (60 MPa and 90 MPa) and temperatures (320 K and 340 K) to calculate the pressure gradient and activation energy of water permeation [24], respectively. Although the selected experimental conditions used were not practical, they were employed to expediate the simulations and reveal the differences in the simulation results regarding the different ZIF-8 defective membranes, all within a realistic time frame with limited computing resources [20,33,34].

The force  f required to act on the C atoms to obtain a given pressure difference is given by

(1) $$f=\frac{\Delta PL_{y}L_{z}}{n}$$

where *n* represents the number of C atoms in the graphene piston, and *L_y_L_z_* describes the piston area. The simulation was subsequently performed for another 10 ns at a timestep of 1 fs while data was recorded simultaneously. Each simulation was repeated twice with a temperature difference of 1 K, and the average data was calculated.

The salt rejection rate *R* is defined as

(2) $$R=1-\frac{N_{\mathrm{permeate}}}{N_{\mathrm{feed}}}$$

where $N_{\mathrm{permeate}}$ denotes the number of ions in the permeate chamber at the final timestep and $N_{\mathrm{feed}}$ denotes the number of ions in the feed chamber at the initial timestep. As the current study addresses only the initial stage of RO desalination, the salt rejection rate was expected to be close to 100% in such an ideal setting.

To calculate the water flux *F* through the nanochannel, the following formula was used:

(3) $$F=\frac{N_{f}-N_{t}}{A(t_{f}-t_{t})}$$

where $N_{f}$ represents the number of water molecules in the permeate chamber at the end of the simulation; $N_{t}$ denotes the lowest number of water molecules in the permeate chamber; $t_{f}$ represents the final timestep; $t_{t}$ is the timestep at which the number of water molecules was the lowest in the permeate chamber and *A* represents the cross-sectional area of the membrane.

## 3. Results and Discussion

As mentioned above, the lattice parameters of the defective ZIF-8 unit cells were expected to differ from those of the pristine ZIF-8 unit cell, and such differences arise from the type and concentration of defect sites. Despite the presence of substitutional defects, the defective ZIF-8 unit cells were assumed to be capable of retaining their pore structures. Zeo++ [35,36] was selected to measure the pore diameters of the largest included sphere (cavity diameter) and the largest free sphere (aperture diameter), which are essential in identifying the relations between the diameters of the spacings and the RO performance of the membranes.

The pristine ZIF-8 unit cell was selected as a point of reference to assess the reliability of the halving method applied during geometric optimization, and the computed values of the unit cell length (17.05 Å), included sphere (11.47 Å), and free sphere (3.40 Å) diameters were found to be consistent with those obtained from experimental studies (16.99 Å [1], 11.6 Å [1,5], and 3.40 Å [1,5], respectively). Therefore, the halving method was considered a reliable approach for obtaining values of the pore diameters and the unit cell lengths.

It is evident that the defective ZIF-8 unit cells are generally smaller than the pristine ZIF-8 unit cells (Table S1). Meanwhile, the cavity diameters (largest included sphere) of the membranes decrease in the following order: pristine > linker1 > linker2 > linker3 > Zn1 > Zn3 > Zn2. Such a situation can be attributed to the loss of functional groups (Zn and linker defects) resulting in the partial collapse of the defective unit cells, thereby causing them to shrink. However, the aperture diameters (largest free sphere) were observed to decrease in the following order: linker3 > linker2 > Zn3 > linker1 > pristine > Zn1 > Zn2. The relatively large aperture diameters of the ZIF-8 cells with missing linkers are due to them possessing fewer linker functional groups, which are bulky and responsible for forming the aperture pores [11,37]. Such a trend is in good agreement with the study of Han et al., which highlighted that a ZIF-8 unit cell containing one linker defect possesses a larger aperture diameter (3.91 Å) than the pristine ZIF-8 (3.44 Å) unit cell [12]. Overall, the pore diameters (aperture and cavity diameters) of the different ZIF-8 membranes in this study are sufficiently large for water molecules (2.8 Å) [38] to pass through.

### 3.1. Salt Rejection

**Figure 2:**
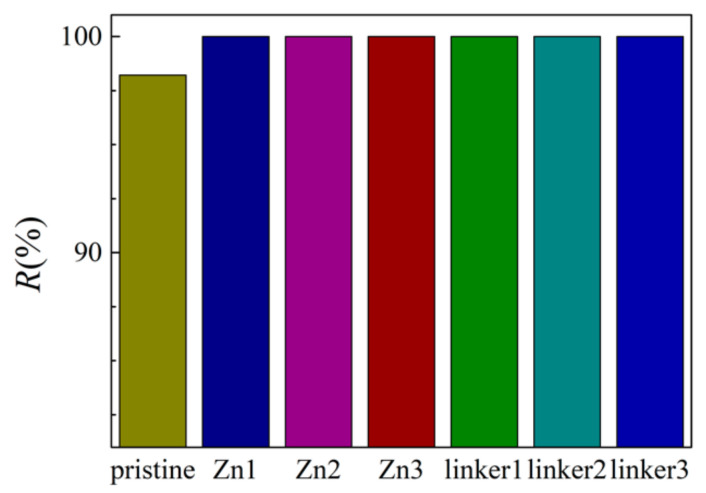
Salt rejection rate of the ZIF-8 membranes with different types and concentrations of substitutional defect sites.

The salt rejection rates for the respective membranes in this study are presented in Figure 2. At an external pressure of 30 MPa, all the defective membranes exhibited complete salt rejection (100%), but the pristine ZIF-8 membrane did not (98.2%). This observation can be explained by the pristine ZIF-8 (11.5 Å) membrane possessing one of the two largest cavity diameters among all the membranes in this study, which may allow the ions to pass through it and enter the permeate region easily. While the ion rejection rate of the pristine ZIF-8 membrane in our study is lower than the value obtained by Hu et al. [5], it should be noted that in their research, 0.5 M NaCl was employed with a fully rigid membrane, which can restrict ion movement. Additionally, only Cl^−^ ions were present in the permeate solution, which suggests that atomic interactions between the ions and the membranes may restrict the movement of Na^+^ ions. Such a proposition will be elaborated later.

**Figure 3:**
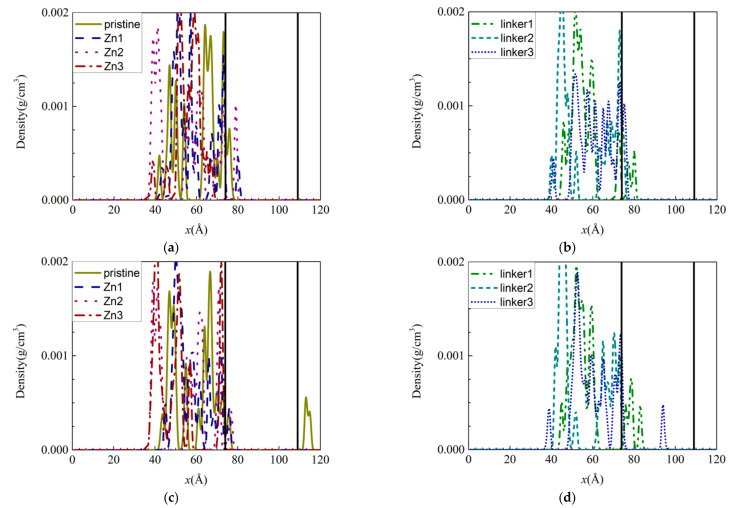
Density distributions of the ions along the *x*-axis inside the ZIF-8 membranes at the end of the simulation under the default operating conditions of 300 K: (**a**,**c**) Na^+^ and (**b**,**d**) Cl^−^ ions. The two ends of the membrane are delineated by vertical black solid lines.

To study the presence of salts within the ZIF-8 membranes, the density distribution graphs of the individual ions were plotted for the respective ZIF-8 membranes (Figure 3). With respect to the distribution of Na^+^ and Cl^−^ ions within the membranes, the Na^+^ ions were highly concentrated just below the surface of the membrane on the feed side, while the Cl^−^ ions were relatively uniformly distributed within the membrane. Based on Figure 3d, Cl^−^ ions were embedded deep in the linker1 and linker3 membranes, and they were present in the permeate regions of the pristine ZIF-8 membrane, as mentioned above. While a few Cl^−^ ions were able to enter the membranes, most ions were unable to do so, and they remained outside the membranes in high concentrations due to the size exclusion effect. By comparing the hydrated radii of the ions (Na^+^ = 2.76 Å [39] and Cl^−^ = 3.3 Å [40]) to the aperture diameters (Table S1), one could deduce that the ions can enter the apertures by losing their hydration shells, since the aperture diameters are larger than the ionic radii (Na^+^ = 0.95 Å [39] and Cl^−^ = 1.81 Å [41]).

Generally, the lack of ions within the Zn substitutional defective membranes can be attributed to their small pore (aperture and cavity) diameters, which render it difficult for the ions to enter the membranes and occupy their interiors. Therefore, only some of the ions managed to occupy the region directly below the surfaces of the membranes. Despite the linker-defective membranes possessing relatively larger pores, most of the Na^+^ ions remained on their surfaces. As a corollary, only a few Na^+^ ions were located directly below the membrane surfaces. Based on the average velocities of the different ions in the ZIF-8 membranes, the Na^+^ ions were mostly faster than the Cl^−^ ions as a result of their smaller mass and size. Thus, the Na^+^ ions can move more rapidly than the Cl^−^ ions within the membranes. However, Cl^−^ ions were present deep within the linker1 and linker3 membranes, as mentioned above, which indicates that atomic interactions between the ions and the ZIF membranes may influence ion movement in the membranes.

To further investigate the movement of ions in the different ZIF-8 membranes, the radial distribution functions (RDFs) of the ions–O (oxygen of water), ZIF–O, and ZIF–ion interactions were plotted. The RDF is defined as the probability of finding two atoms separated at a specific distance from each other. Therefore, if the *r* values of the ZIF–ion interactions are smaller than those of the ion–O interactions, there will be a high possibility of membrane adsorption due to a greater affinity between the ZIFs and the ions [20]. In addition, the average interaction energies of the ZIF–ion and ZIF–O interactions throughout the entire simulation were also calculated and plotted [42]. The more negative an interaction energy value is, the stronger the interaction will be, which corresponds to stronger adsorption of ions and water molecules by the atoms constituting ZIF-8.

**Figure 4:**
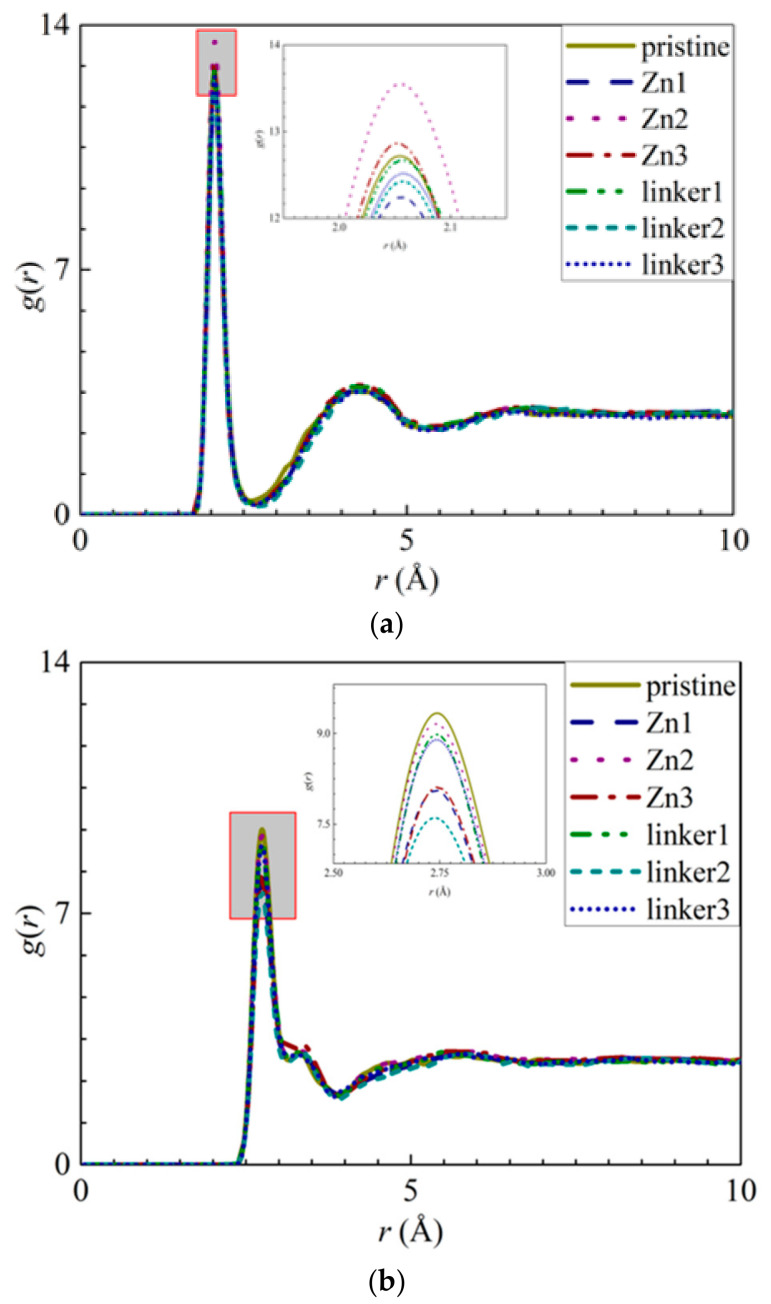
RDFs of the O atoms from the water molecules around the ions: (**a**) Na^+^ ions and (**b**) Cl^−^ ions for the respective ZIF-8 membranes.

The appearance of the first peaks in the RDF of the ion–O interactions (Figure 4) indicates the formation of the first hydration shell around the ions, and the values of *r* for the first peak for the Na^+^ and Cl^−^ ions were reported as 2.05 Å and 2.75 Å, respectively. The first peak height of the Na^+^ ions decreases in the following order: Zn2 > Zn3 > pristine > linker1 > linker3 > linker2 >Zn1, while the first peak height of the Cl^−^ ions decreases in the following order: pristine > Zn2 > linker1 > linker3 > Zn3 > Zn1 > linker2. The hydration level of the Cl^−^ ions was found to be higher than that of the Na^+^ ions, which is in agreement with the results of Dahanayaka et al. [34] These observations imply that the hydration levels of the Na^+^ and Cl^−^ ions are affected by the type and concentration of defects in the ZIF-8 membrane.

**Figure 5:**
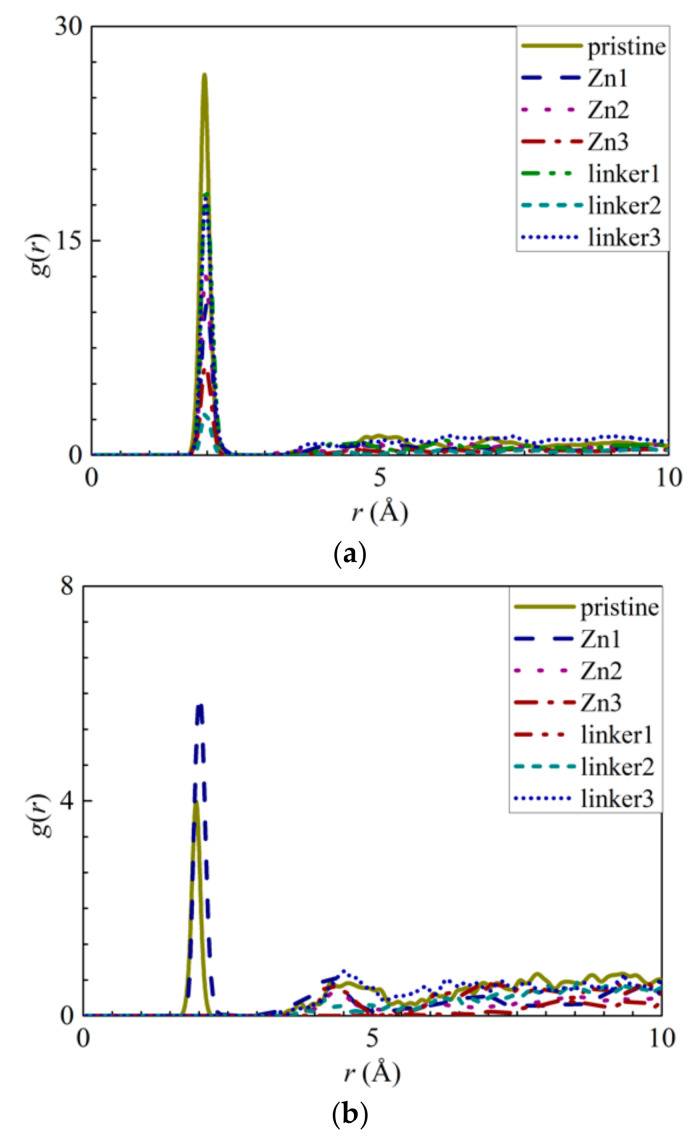
RDFs of the respective ZIF-8s: (**a**) Na^+^ ions around the nitrogen atoms and (**b**) Cl^−^ ions around the Zn atoms.

The interactions between the atoms from the ZIF-8 membranes and the ions were explored by calculating their RDFs and the interaction energies between the ions and water molecules with the ZIF atoms. The RDF results of the pristine ZIF-8 membrane indicate that the positively charged Na^+^ ions and the negatively charged Cl^−^ ions interact strongly with the N (Figure 5a) and Zn (Figure 5b) atoms, respectively. The interaction energy of the N–Na^+^ pairs (Figure S4a) decreases in magnitude in the following order: Zn2 > pristine > linker3 > Zn1 > linker2 > linker1 > Zn3. This implies that the adsorption of Na^+^ ions is the strongest and weakest in the Zn2 and Zn3 membranes, respectively. According to the graph in Figure S4b, the magnitude of the Zn–Cl^−^ interaction energy decreases in the following order: linker3 > pristine > Zn2 > Zn1 > linker2 > linker1 > Zn3, with linker3 and Zn3 membranes exhibiting the strongest and weakest affinity toward Cl^−^ ions, respectively. However, based on the RDF graph in Figure 5b, aside from the Zn1 and pristine ZIF-8 membranes, the other defective ZIF-8 membranes did not display distinct curves. This could be attributed to the ZIF-8 membranes exhibiting a stronger affinity toward Na^+^ than Cl^−^ ions, which is evident by the Na^+^ ions possessing higher RDF peak values and greater interaction energies than the Cl^−^ ions. Hence, it is possible to dampen ion adsorption on a ZIF-8 membrane by introducing Zn substitutional defects, as evidenced by the Zn3 membrane exhibiting the weakest ion adsorption at its Zn and N sites. However, it is also possible to improve ion adsorption on ZIF-8 membranes by increasing the number of linker defect sites within them.

**Figure 6:**
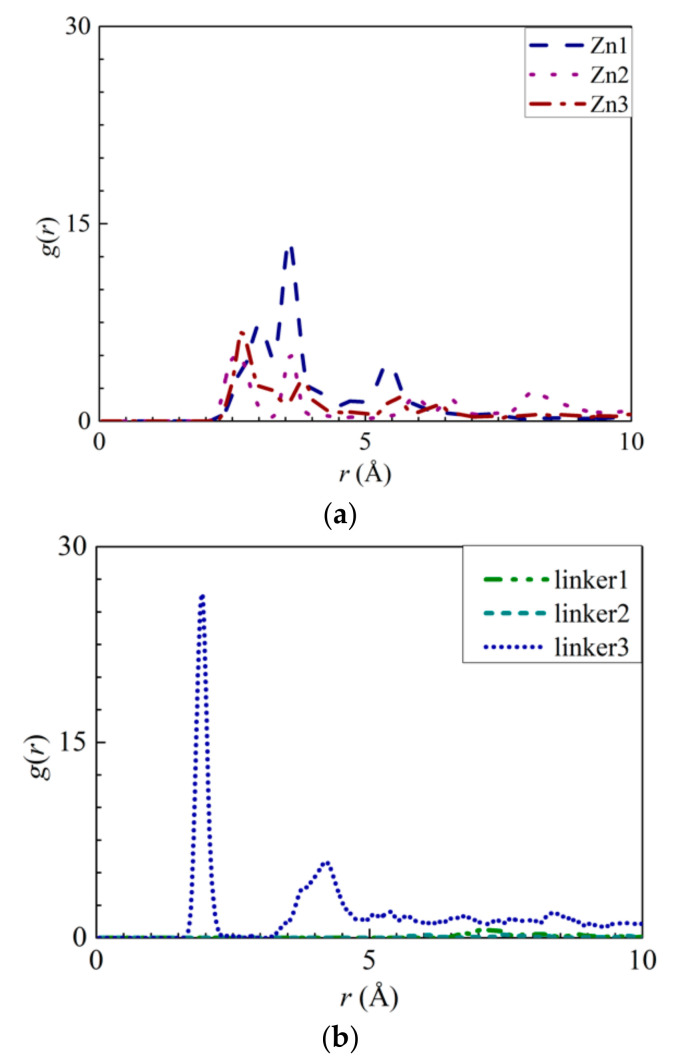
RDFs of the Na^+^ ions around the atoms from the respective ZIFs: (**a**) H3 atoms at the Zn substitutional defect sites and (**b**) O2 atoms at the linker substitutional defect sites, respectively.

**Figure 7:**
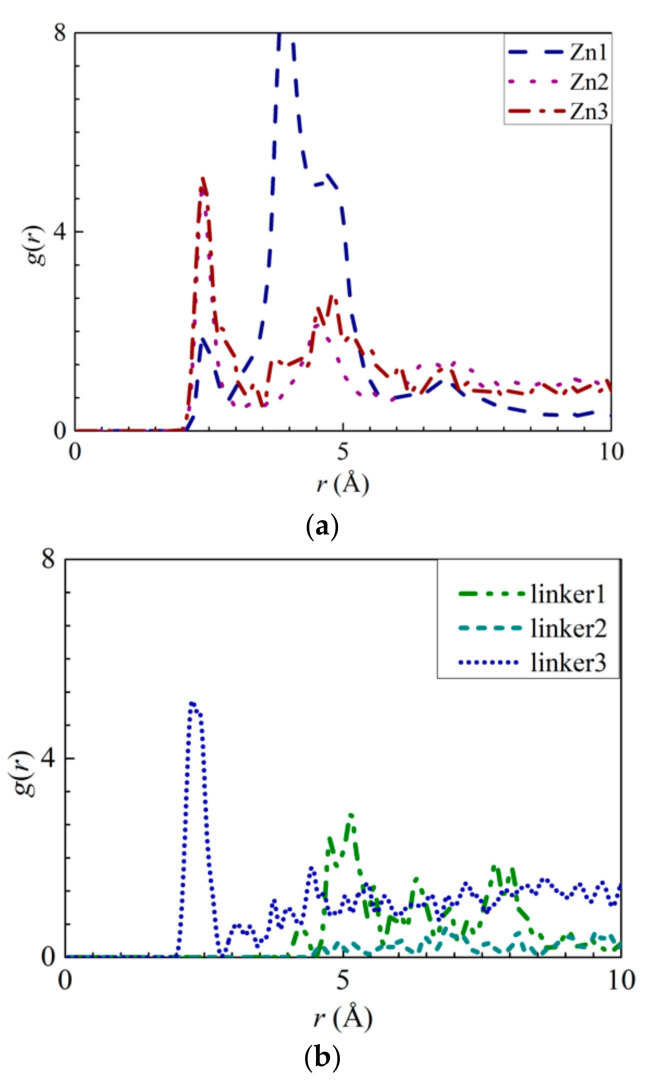
RDFs of the Cl^−^ ions around the atoms from the respective ZIFs: (**a**) H3 atoms at the Zn substitutional defect site and (**b**) H3 atoms at the linker substitutional defect site, respectively.

To explore the possibility of ion adsorption occurring at the defect sites, the RDF curves of the atoms in the defect sites with respect to the solute ions were plotted (Figure 6 and Figure 7). Furthermore, the interaction energies between the ions and the atoms were measured (Figures S4a,b, S5a, S6a, S7a, and S8a). By analyzing Na^+^ adsorption at the Zn substitutional defect sites, the first peak height of the H3–Na^+^ curve was observed to decrease in the following order: Zn3 > Zn1 > Zn2, with the first RDF peaks of the Zn3 and Zn2 membranes corresponding to a common *r* value of 2.65 Å, and the first RDF peak of Zn1 being located at 3.55 Å. Unlike the H3–Na^+^ interactions, the N–Na^+^ interactions are sufficiently strong enough to overcome the Na^+^–O interactions, which indicates that the Zn defects are not involved in Na^+^ adsorption. This is also evident from the positive interaction energy values for H3–Na^+^ pairs, indicating that no Na^+^ adsorption had taken place. Therefore, the H3 atoms’ peak value in the Zn defects can be attributed to the proximity of the H3 atoms to the N atoms. For linker defects, only the O2 atoms in the linker3 membrane showed distinct interactions with Na^+^. This is also supported by Figure S8a, which shows the Na^+^ adsorption taking place at the O2 atoms of the three ZIF-8 membranes with linker defects. The lack of well-defined curves in the RDF diagrams of the linker1 and 2 membranes could be due to the huge differences between their interaction energies and that of the linker3 membrane. Meanwhile, both the *r* values of O2–Na^+^ (Figure 6b) and N–Na^+^ (Figure 5a) interactions are equal at 1.95 Å. However, their respective interaction energy graphs (Figures S4a and S8a) reveal that the N–Na^+^ pairs exhibit a higher interaction energy than the O2–Na^+^ pairs. The stronger N–Na^+^ interactions may have also dampened the O2–Na^+^ interactions, which may explain the lack of distinct curves in the RDF diagrams of the linker1 and linker2 membranes.

As for Cl^−^ adsorption at the defect sites, the first peak height of the H3–Cl^−^ (Figure 7a) curves in the Zn defective membranes decreases in the following order: Zn2 > Zn3 > Zn1 at 2.35 Å. Such a trend is similar to the pattern exhibited by the interaction energy graphs (Figure S5a) in which they decreases in the same order. Therefore, there exists an ideal number of Zn defect sites per unit cell for optimal Cl^−^ adsorption. For the linker defective membranes, only the linker3 membrane exhibits a distinct curve for H3–Cl^−^ interactions, which suggests that only the H3 atoms within it can adsorb Cl^−^ ions. However, based on the interaction energy graphs (Figures S6a and S7a), involving the H3 and H4 atoms, Cl^−^ adsorption occurred in the cases of the three ZIF-8 membranes with linker defects. The discrepancy between the RDF results and the interaction energy graphs can be attributed to the difference in the interaction energy values between the linker3 membrane and the other two linker defective membranes (linker1 and linker2). Since they correspond to relatively weak Cl^−^ adsorption, the RDF curves of the linker1 and linker2 membranes do not exhibit distinct peaks (Figure 7). Therefore, even if Cl^−^ ions could be potentially adsorbed onto the H3 and H4 atoms of the linker1 and linker2 membranes, such an effect may not be sufficiently strong to overcome the Cl^−^–O interactions. Since the *r* values of the H3–Cl^−^ interactions in the defect sites of the Zn2, Zn3, and linker3 membranes are smaller than those of the Cl^−^–O interactions, the defect sites of these defective membranes can facilitate Cl^−^ adsorption.

Based on the interaction energy graphs (Figures S5a, S6a, S7a, and S8a), all the atoms except the O2 atoms from the linker defect sites can facilitate Cl^−^ adsorption, while the O2 atoms are capable of can adsorbing Na^+^ ions. Although ion adsorption is weaker at the defect sites than at the Zn and N atoms, it is possible to improve ion adsorption in ZIF-8 membranes by introducing linker substitutional defects. In RO applications, since ZIF-8 membranes are constantly in contact with water, more linker defects can be expected to be formed, which in turn enhances ion adsorption on the membranes without involving any additional modifications.

According to the different RDF curves, the ZIF-8 membranes exhibit a higher affinity toward Na^+^ ions than Cl^−^ ions. As a result of such a relatively high affinity, the Na^+^ ions could only occupy a few angstroms below the surfaces of the membranes despite being smaller than the Cl^−^ ions. Meanwhile, based on the RDF results, the pristine ZIF-8 membrane has the highest affinity toward Cl^−^ ions among all the membranes. Despite their relatively low Cl^−^ affinity, the defective ZIF-8 membranes may also facilitation Cl^−^ adsorption. However, such weak Cl^−^ ion adsorption implies that while Cl^−^ ions can still be adsorbed onto the defective ZIF-8 membranes, they cannot be trapped within the membranes. Instead, the Cl^−^ ions will pass through these membranes despite their relatively large ionic radius.

### 3.2. Water Flux

**Figure 8:**
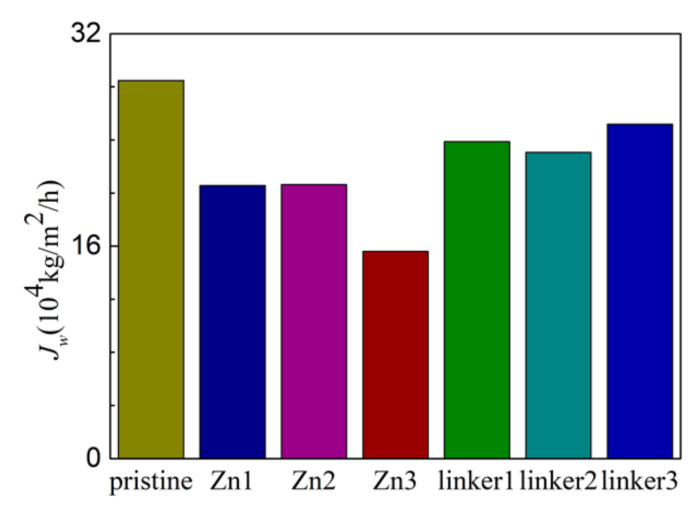
Water flux of the ZIF-8 membranes with different types and concentrations of substitutional defect sites.

In RO, water flux across the different ZIF-8 membranes (Figure 8) is affected by the size of the pore diameters, the activation energy for water permeation, ZIF hydrophilicity, and the ordering of water molecules in the entire system. The water flux values at different pressures in this study are higher than those obtained in the ZIF-8 study by Hu et al. [5], which can be attributed to Hu et al. employing a rigid ZIF-8 membrane in their study [5], while this study utilized more flexible membranes.

**Figure 9:**
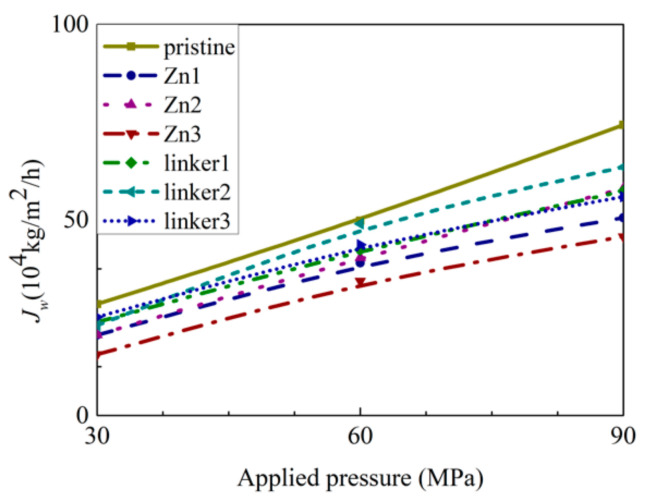
Dependence of water flux *J_W_* on the applied pressure for the respective ZIF-8 membranes.

As shown in Figure 8, the pristine ZIF-8 membrane corresponds to the highest water flux, followed by the linker substitutional defective membranes (linker3, linker1, and linker2) and the Zn substitutional defective membranes (Zn1, Zn2, and Zn3). As evident from the relationship between the pore diameter and the water flux, large cavities and apertures increase the water flux, as evident in the cases of the pristine ZIF-8 membrane that possesses the largest cavity diameter and exhibit the highest water flux and the linker3 membrane that possesses the largest aperture diameter and exhibit the second-highest water flux. With respect to the type of defects, the ZIF-8 membranes with linker defects generally exhibit the second-highest water flux due to them possessing larger cavities than the Zn defects and the largest aperture diameters collectively. However, even if the cavities or the apertures are large, the water flux will still be low if the other kind of pores are too small. This is evident in the case of the Zn3 membrane (3.52 Å), which despite having a larger aperture diameter than pristine ZIF-8 membrane (3.40 Å), possesses the second smallest cavity diameter (11.0 Å) among the studied ZIF-8 membranes. Based on Figure 9, the pristine ZIF-8 membrane displays the highest water flux among all the membranes at higher pressure values. Furthermore, under an applied pressure of 60 MPa, the ZIF-8 membranes with linker substitutional defects (linker1, linker2, and linker3) generally exhibit the second-highest water flux after the pristine ZIF-8 membrane.

**Figure 10:**
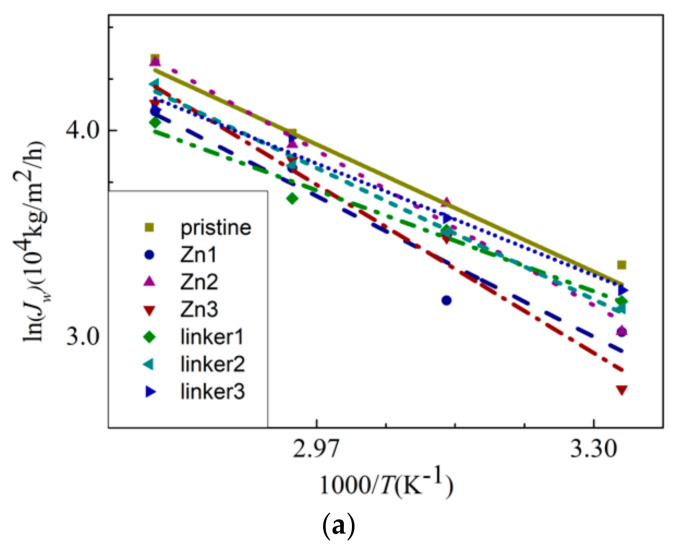

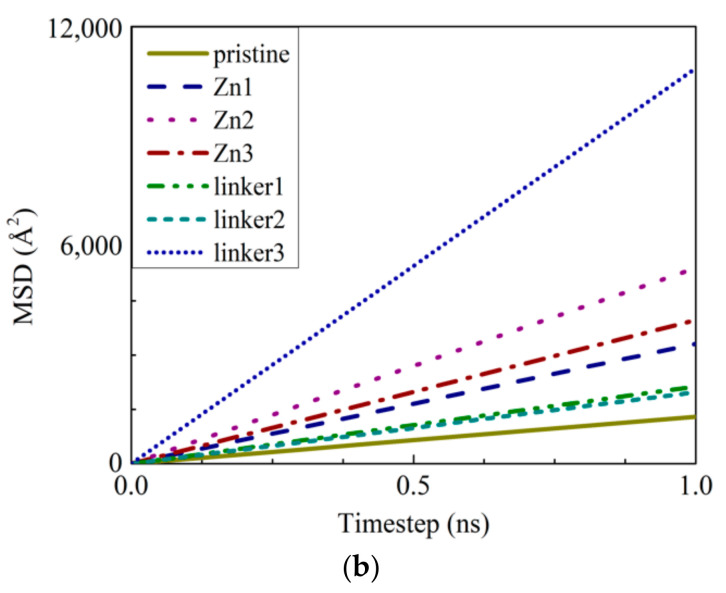
(**a**) The Arrhenius plots of the water flux *J_W_* and (**b**) MSDs of the water molecules inside the feed solution.

To explore the temperature dependency of water flux through the membranes, the Arrhenius equation [43] was invoked. It is defined as:

(4) $$J_{W}=e^{\frac{-E_{a}}{RT}}$$

where *E*_a_, R, and *T* are the apparent activation energy, gas constant, and temperature, respectively. The water flux *J_W_* against the reciprocal of *T* is plotted in Figure 10a, and the linear fitting of the curves provides the apparent activation energy values for water permeation through the membranes. From the gradients of the Arrhenius plots for the respective ZIF-8 membranes, the activation energy value for water permeation decreases in the following order: Zn3 (20.5 kJ/mol) > Zn2 (18.9 kJ/mol) > Zn1 (17.2 kJ/mol) > linker2 (16.0 kJ/mol) > pristine (15.5 kJ/mol) > linker3 (13.6 kJ/mol) > linker1 (12.3 kJ/mol). Positive values of the activation energy indicate that water flux can be improved with a higher feed temperature [23]. Based on the values of *E*_a_, the presence of Zn substitutional defects in a ZIF-8 membrane can generally increase the value of *E*_a_, while it is possible for linker substitutional defects to lower the value of *E*_a_, with the latter depending on the concentration of linker substitutional defects in the membrane. Therefore, water flux may not improve significantly for ZIF-8 membranes with Zn substitutional defects even by increasing the feed temperature. According to the value of *E*_a_ of the pristine ZIF-8 membrane, its corresponding activation energy is lower than that recorded in the study of Hu et al. (24.4 kJ/mol) [5], in which used a fully rigid ZIF-8 membrane that hinders water flow was utilized, as mentioned above.

To study the diffusion of water through the membranes, the mean square displacements (MSDs) of the water molecules were evaluated (Figure 10b). The measured displacements of the water molecules were measured with respect to their equilibrium positions. The linear fitting of an MSD $\langle \Delta s^{2}\rangle \left(t\right)$ curve provides the diffusion coefficient *D* according to the relation $\langle \Delta s^{2}\rangle \left(t\right)\approx 6Dt$, where *t* is the simulation time. This approximation method is commonly used in MD simulations to obtain the membrane diffusion coefficients [23]. Based on our results, the value of *D* decreases in the following order: linker3 > Zn2 > Zn3 > Zn1 > linker1 > linker2 > pristine, which indicates that water molecules moved more quickly in substitutional defective ZIF-8 membranes than pristine ZIF-8 membranes. However, such an observation appears to contradict the water flux records, which show that the presence of substitutional defects reduces the water flux through a ZIF-8 membrane. Therefore, the obtained diffusion coefficient values suggest that the rate of diffusion does not depend on the size of the cavities or apertures. Instead, it depends on the interactions between the water molecules and the constituent atoms of the ZIF-8 membranes, as described in the subsequent paragraphs.

**Figure 11:**
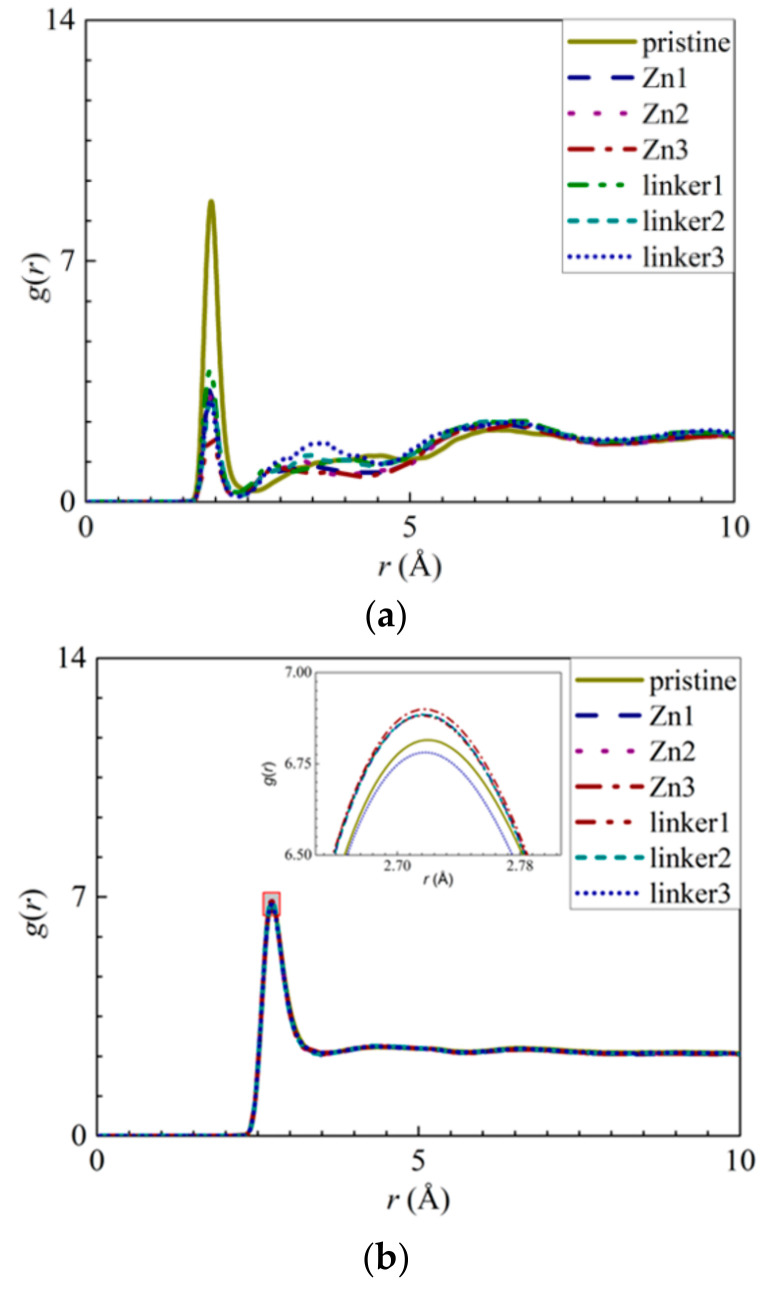
RDFs of the O atoms of water molecules surrounding the respective atoms: (**a**) Zn atoms and (**b**) other O atoms of water molecules. The inset in (**b**) presents an enlarged view of the first peak, as delineated by the shaded rectangle.

Another factor that contributed to the reduced water flux in the linker substitutional defect membranes is their enhanced hydrophilicity, which was caused by the loss of hydrophobic organic linkers and the oxygenation of the ZIF-8 membranes. The hydrophilicity of ZIF-8 membranes can be assessed according to their RDFs, which is necessary to analyze the interactions between the membrane atoms and the water molecules. As the Zn atoms in the pristine ZIF-8 membranes exhibit the strongest interactions with the O atoms in the water molecules, the RDFs and the interaction energies of Zn–O atom pairs (Figure 11a and Figure S4c) from the different ZIF-8 membranes were compared. The heights of the first peaks of the Zn–O atom pairs at an *r* value of 1.95 Å decrease in the following order: pristine > linker1 > Zn1 > linker2 > Zn2 > linker3 > Zn3, while the magnitudes of the interaction energies decrease in the following order: linker3 > linker2 > pristine > linker1 > Zn1 > Zn2 > Zn3. Both of these trends indicate that the presence of Zn defects will weaken the Zn–O interactions, since the weakened Zn–O interactions in the Zn substitutional defects may be attributed to the decreasing number of Zn atoms in the membranes.

**Figure 12:**
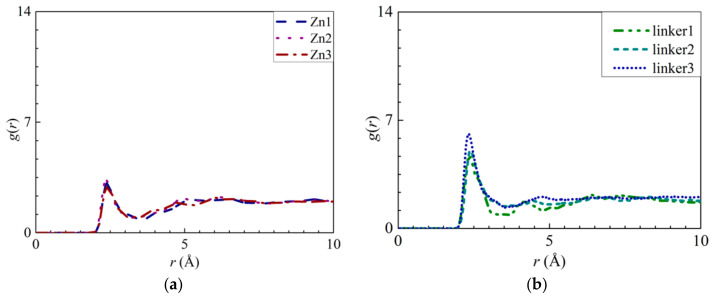

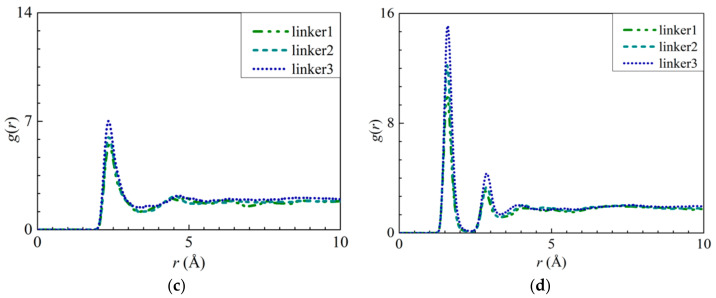
RDFs of the O atoms of water molecules around the atoms at the respective defect sites: (**a**) H3 atoms at the Zn substitutional defect site, (**b**) H3 atoms and (**c**) H4 atoms at the linker substitutional defect site. (**d**) RDF of the hydrogen atoms of water molecules around the O2 atoms at the linker substitutional defect site.

Based on the interactions between the O atoms in the water molecules and the atoms within the defects (Zn and linker), RDF curves and interaction energy graphs of these O atoms were plotted (Figure 12). By analyzing the H3–O interactions in the Zn1, Zn2, and Zn3 membranes (Figure 12a), the heights of the peaks at an *r* value of 2.35 Å decrease in the following order: Zn2 > Zn1 > Zn3. The negative values from the interaction energy graphs (Figure S5b) indicate that by increasing the number of Zn defect sites, the strength of the H3–O interactions can be improved. However, these interactions are not as strong as the Zn–O interactions, which that Zn substitutional defects could not improve ZIF-8 hydrophilicity.

In a linker substitutional defect, all three atoms (H3, H4, and O2) that replace the missing linker exhibit distinct peaks in their RDFs with respect to the O and H atoms from the water molecules. For the H3–O, H4–O, and O2–H interactions, the first peaks correspond to *r* values of 2.35, 2.35, and 1.55 Å, respectively, and their heights all decrease in the following order: linker3 > linker2 > linker1. A similar trend can be observed for the corresponding interaction energy graphs (Figures S6b, S7b, and S8b), which could be attributed to the defect sites introducing additional interactions. By comparing the *r* values of the H3–O, H4–O, and O2–H interactions with those of the O–O (2.75 Å) and Zn–O interactions (1.95 Å), the H3–O and H4–O interactions were determined to be weaker than the Zn–O interactions despite the fact that their *r* values are smaller than that of the O–O interactions, which implies that the defect site is hydrophilic in nature. Overall, since the Zn–O interactions correspond to a higher interaction energy than the H3–O, H4–O, and O2–H interactions, the Zn atoms exhibit the strongest interactions with water. 

Furthermore, the heights of the first peaks of the O–O curves (Figure 11b) decrease in the following order: Zn3 > linker2 > Zn1 > Zn2 > linker1 > pristine > linker3, which indicate that the hydrophilicity of a ZIF-8 membrane can be potentially improved by introducing a sufficient amount of linker defects to it. However, such an enhanced hydrophilicity will reduce the water flux through the membrane, as evident from the relatively low water flux through the linker substitutional defective ZIF-8 membranes compared with that of the pristine ZIF-8 membrane. According to the heights of the first peaks of the RDFs pertaining to the interactions between the salt ions and water molecules and the atoms at the defect sites, the H3 atoms at the Zn defect sites (Zn1, Zn2, and Zn3) generally exhibit a higher affinity towards Cl^−^ ions than water molecules. Meanwhile, the H3, H4, and O2 atoms at the linker1, linker2, and linker3 defect sites generally display a stronger affinity toward water molecules than the salt ions.

According to the interaction energy graphs corresponding to the interactions between the salt ions and water molecules and the membrane atoms at the defect sites, all the energy values pertaining to the membrane atoms and the water molecules are several times higher than those regarding the membrane atoms and the ions. As mentioned previously, this phenomenon is due to the simulation systems containing more water molecules than membrane atoms at the defect sites, and thus interactions between the ZIF-8 atoms and the water molecules occurred much more frequently than the interactions between the ZIF-8 atoms and the salt ions.

The results of this study suggest that the RO seawater desalination performance of the pristine ZIF-8 membrane is superior to that of the defective membranes, which is in good agreement with the study of Zhang et al. [10], in which even a small number of defects in a ZIF-8 unit cell significantly affected the performance of the corresponding RO desalination membrane. As mentioned previously, linker substitutional defects are more likely to be formed than Zn substitutional defects. Experimental studies regarding the presence of water-based defects have indicated that the presence of defects within a ZIF-8 membrane allows more water molecules to pass through it, which can be attributed to the structural collapse of ZIF-8 unit cells due to the formation of a large number of defect sites in each unit cell. Such structural collapse results in the loss of large ZIF-8 clusters, which in turn creates large voids within the ZIF-8 membranes. In the scanning electron microscopy [44] images of defective ZIF-8 membranes obtained by Zhang et al. [9], holes are visible within the membranes on a scale of 2 µm and a magnification of ×10,000. As the duration of the ZIF-8 submersion in water was prolonged, the holes were enlarged. Such an observation indicates that within defective ZIF-8 membranes, whole ZIF-8 unit cells are missing instead of individual Zn atoms and/or linkers. Therefore, the formation of water-based substitutional defects within a ZIF-8 unit cell does not contribute significantly to the increase in the water flux of the corresponding ZIF-8 membrane, but it may improve ZIF-8 hydrophilicity of the membrane if sufficient linker substitutional defect sites exist.

## 4. Conclusions

In this study, molecular dynamics simulations were employed to simulate the rejection of ions from seawater by the pressure-driven transport of seawater through seven ZIF-8 membranes with different types and concentrations of substitutional defects. This study investigated the influence of defects in ZIF-8 RO membranes on their RO desalination performance through molecular dynamics simulations. By keeping the driving pressure constant, the desalination performance of the seven ZIF-8 membranes were evaluated in terms of their water flux and ion rejection. The ion density distributions within the membranes and the RDFs of various atomic pairs were also explored to understand the water and ion transport behaviors within the membranes.

Among the seven types of ZIF-8 membranes explored in this study, the pristine ZIF-8 membrane exhibits the best desalination performance in terms of water flux, but it possesses the lowest ion rejection at 98.2%. It was also observed that the positions of the ions with respect to the membranes, i.e., on the surfaces of the membranes or deep within them, are highly dependent on the sizes of the pores and the interactions between the membrane atoms and the ions in the water. Only a small number of Cl^−^ ions remained within the membranes, which can be attributed to the relatively weak interactions between the Cl^−^ ions and the membrane atoms. Generally, the water flux is influenced by the pore sizes (cavity and aperture) and the hydrophilicity of the ZIF-8 membranes, and strong atom–water interactions will reduce the water flux. 

The RDF and interaction energy were parameters employed to determine how the ZIF-8 membranes interact with the ions. Our results indicate that the Na^+^ and Cl^−^ ions are primarily adsorbed by the N and Zn atoms, respectively. While Na^+^ ions can be adsorbed by the O2 atoms in the linker substitutional defects and Cl^−^ ions can be adsorbed by the H3 atoms in the Zn substitutional defects as well as the H3 and H4 atoms in the linker substitutional defects, the ion adsorption at the N and Zn atoms is stronger. Furthermore, the hydrophilicity of a ZIF-8 membrane can be improved by introducing more linker substitutional defects, since the hydrophobic linker is being replaced by a more hydrophilic functional group.

Note that our study focuses on the effects of water-induced substitutional defects in ZIF-8 unit cells at the nanoscale level on the RO desalination performance of ZIF-8 membranes, and it does not account for the loss of ZIF-8 unit cells at the macroscale level. Therefore, the differences in the desalination performance of the various ZIF-8 membranes in this study are attributed to the distinct type and concentration of substitutional defects (Zn and linker) for each membrane. Overall, through molecular dynamics simulations, this research demonstrates that Zn and linker substitutional defects within the unit cells of a ZIF-8 membrane induce changes in its pore (cavity and aperture) diameter, unit cell parameters, hydrophilicity, water flux, and ion rejection. Such a study elucidates the application of molecular dynamics simulations in predicting the type and concentration of defect sites required to obtain the optimal pore diameters and membrane hydrophilicity to maximize the water flux and ion rejection of ZIF-8 RO membranes.

## Data Availability

The data presented in this study are available in DOI: https://doi.org/10.21979/N9/AVCQMD (accessed on 15 March 2021).

## Supplementary Materials

The following are available online, Figure S1: Hydrolysis reaction of ZIF-8 with water molecules to form defect sites: (a) Zn substitutional defect site and (b) linker substitutional defect site. V_Zn and L denote the Zn substitutional and linker, respectively, Figure S2: Atom representations of ZIF-8s with different defects: (a) Zn substitutional defects (H3) and (b) linker substitutional defects (O(H4)-H3-O(H4)) with respect to the ZIF-8 structure, Figure S3: Representative unit cell of defective ZIF-8s: (a) one Zn substitutional defect site, Zn1, (b) two Zn substitutional defect sites, Zn2, (c) three Zn substitutional defect sites, Zn3, (d) one linker substitutional defect site, linker1, (e) two linker substitutional defect sites, linker2 and (f) three linker substitutional defect sites, linker3, Table S1: Cell lengths, cavity diameters and aperture diameters of pristine, Zn1, Zn2, Zn3, linker1, linker2 and linker3, respectively obtained from Zeo++, Table S2: Partial charges of the atoms in the respective ZIF-8 unit cells, Figure S4. Interaction energy graphs of the respective ZIF-8s: (a) N atoms with the Na+ ions, (b) Zn atoms with the Cl^−^ ions, and (c) Zn atoms with the O atoms of water molecules, Figure S5: Interaction energy graphs of the respective ZIF-8s with number of Zn substitutional defect sites: (a) H3 atoms with the Cl^−^ ions and (b) H3 atoms with the O atoms of water molecules, Figure S6: Interaction energy graphs of the respective ZIF-8s with number of linker substitutional defect sites: (a) H3 atoms with the Cl^−^ ions and (b) H3 atoms with the O atoms of water molecules, Figure S7: Interaction energy graphs of the respective ZIF-8s with number of linker substitutional defect sites: (a) H4 atoms with the Cl^−^ ions and (b) H4 atoms with the O atoms of water molecules, Figure S8: Interaction energy graphs of the respective ZIF-8s with number of linker substitutional defect sites: (a) O2 atoms with the Na+ ions and (b) O2 atoms with the H atoms of water molecules.
