# Supplementary material for: Influence of Substitutional Defects in ZIF-8 Membranes on Reverse Osmosis Desalination: A Molecular Dynamics Study
Source: Molecules. 2021 Jun 3;26(11):3392. doi: 10.3390/molecules26113392 (PMC8200035; doi:10.3390/molecules26113392)
Supplement: Supplementary file 1 [file molecules-26-03392-s001.zip › molecules-1206544-supplementary.pdf]

Supporting Information For:

Influence of substitutional defects in ZIF-8 membranes on reverse osmosis desalination: A molecular dynamics study

1. Formation of water-based substitutional defects in ZIF-8.

(a)

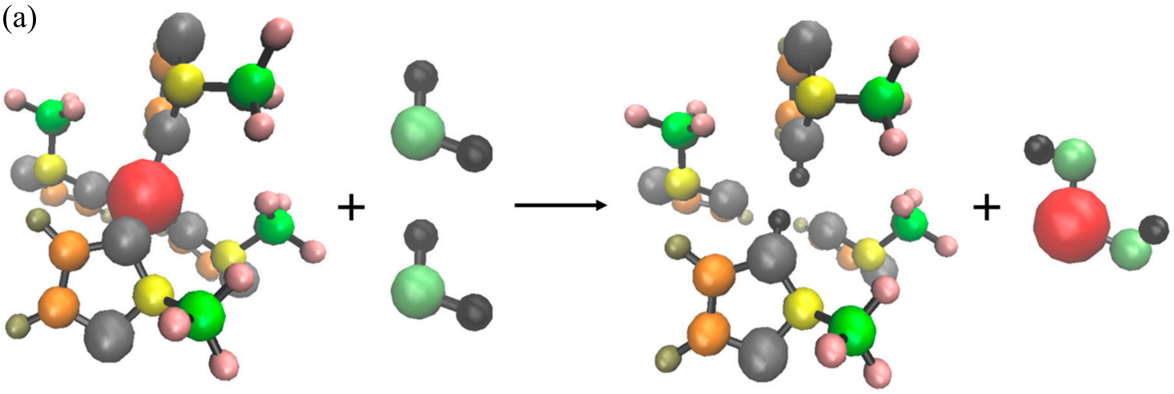

$$\mathrm{ZIF-8_{(s)} + 2H_2O_{(l)} \rightarrow ZIF-8[V_{Zn}]_{(s)} + Zn(OH)_{2(s)}}$$

(b)

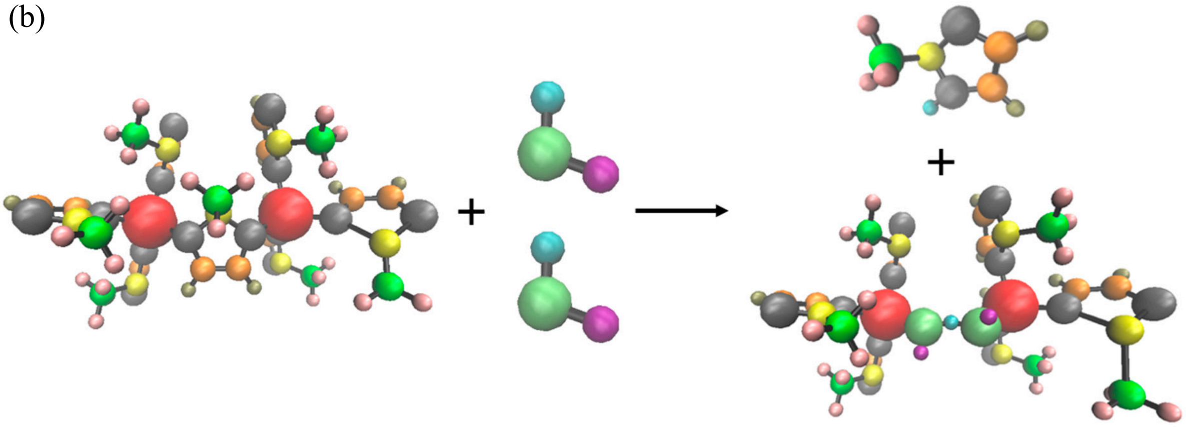

$$\mathrm{ZIF-8_{(s)} + 2H_2O_{(l)} \rightarrow ZIF-8[Zn-OH-H-OH-Zn]_{(s)} + HL_{(g)}}$$

Figure S1. Hydrolysis reaction of ZIF-8 with water molecules to form defect sites: (a) Zn substitutional defect site and (b) linker substitutional defect site. V_{Zn} and L denote the Zn substitutional and linker, respectively.

2. Representations of the different atoms in the defects with respect to the ZIF-8.

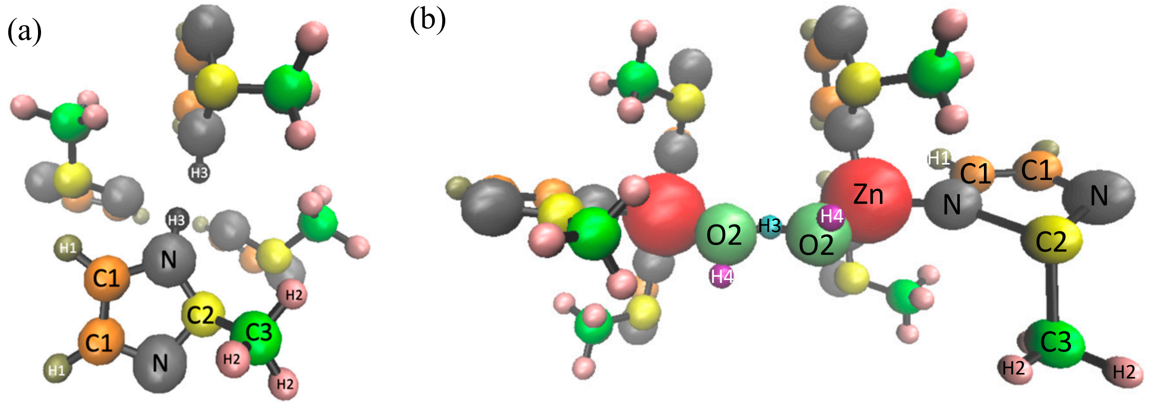

Figure S2. Atom representations of ZIF-8s with different defects: (a) Zn substitutional defects (H3) and (b) linker substitutional defects (O(H4)-H3-O(H4)) with respect to the ZIF-8 structure.

3. ZIF-8 defective unit cells used to construct the RO membranes.

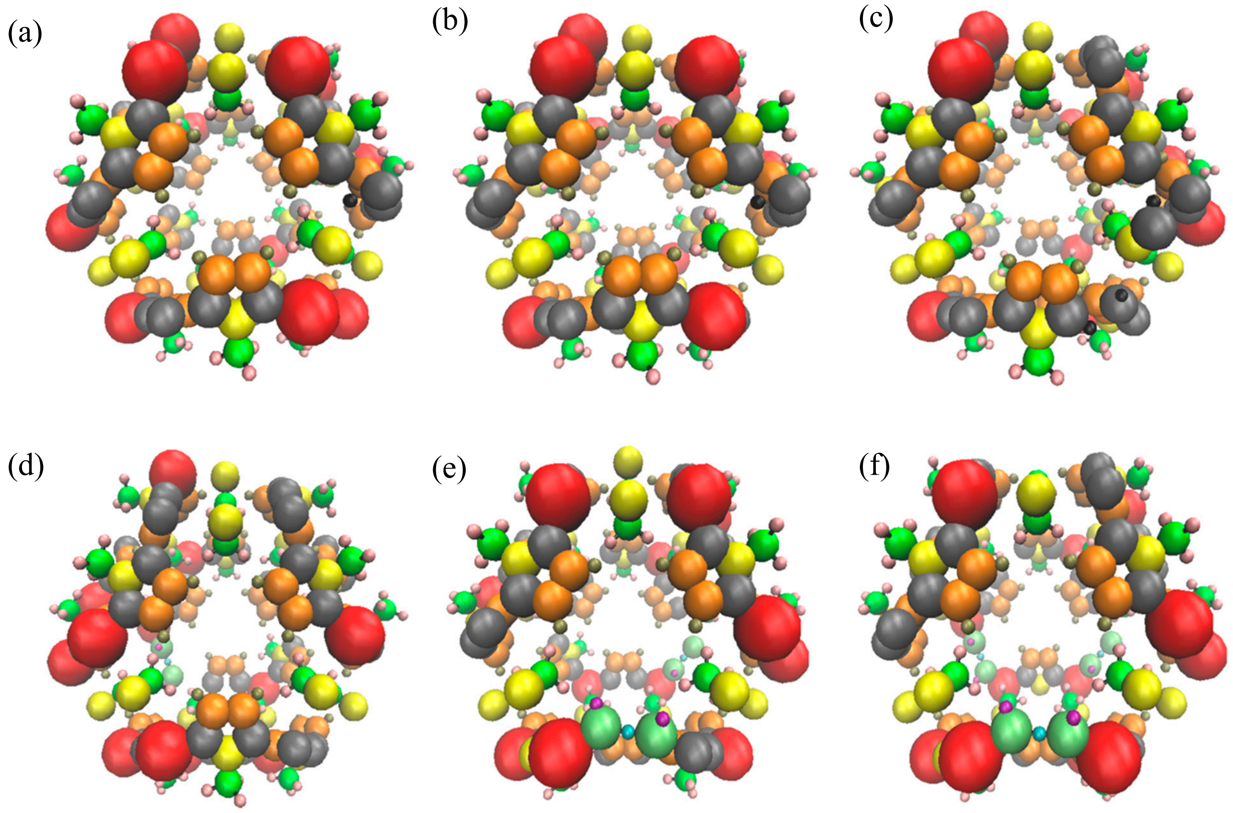

Figure S3. Representative unit cell of defective ZIF-8s: (a) one Zn substitutional defect site, Zn1, (b) two Zn substitutional defect sites, Zn2, (c) three Zn substitutional defect sites, Zn3, (d) one linker substitutional defect site, linker1, (e) two linker substitutional defect sites, linker2 and (f) three linker substitutional defect sites, linker3.

4. Properties of the representative ZIF-8 unit cells.

Table S1. Cell lengths, cavity diameters and aperture diameters of pristine, Zn1, Zn2, Zn3, linker1, linker2 and linker3, respectively obtained from Zeo++.

ZIF-8	Cell length (Å)	Cavity diameter (Å)	Aperture diameter (Å)
**pristine**	17.05	11.47	3.40
**Zn1**	16.95	11.32	3.34
**Zn2**	16.94	11.00	3.36
**Zn3**	17.02	11.02	3.52

**linker1**	17.07	11.47	3.48
**linker2**	16.95	11.35	3.59
**linker3**	17.02	11.34	3.83

5. **Partial charges of the respective ZIF-8 unit cells.**

Table S2. Partial charges of the atoms in the respective ZIF-8 unit cells.

	pristine	Zn1	Zn2	Zn3	linker1	linker2	linker3
**Zn**	1.1681	1.1679	1.1672	1.1680	1.1743	1.1811	1.1868
**N**	-1.2491	-1.2414	-1.2449	-1.2457	-1.2392	-1.2429	-1.2432
**C1**	0.3442	0.3421	0.3343	0.3387	0.3340	0.3329	0.3348
**C2**	0.9566	0.9623	0.9654	0.9648	0.9479	0.9696	0.9614
**H1**	0.0783	0.0757	0.0838	0.0832	0.0841	0.0824	0.0849
**C3**	-0.0575	-0.0751	-0.0689	-0.0671	-0.0599	-0.0710	-0.0586
**H2**	0.0566	0.0595	0.0587	0.0579	0.0569	0.0579	0.0540
**H3**	-	0.5525	0.5694	0.5529	0.6485	0.6579	0.6469
**H4**	-	-	-	-	0.5846	0.5940	0.5890
**O2**	-	-	-	-	-1.2449	-1.2587	-1.2484

6. Interaction energy graphs between the ions and water molecules with the Zn and N atoms from the different ZIF-8 membranes with different types and number of defects.

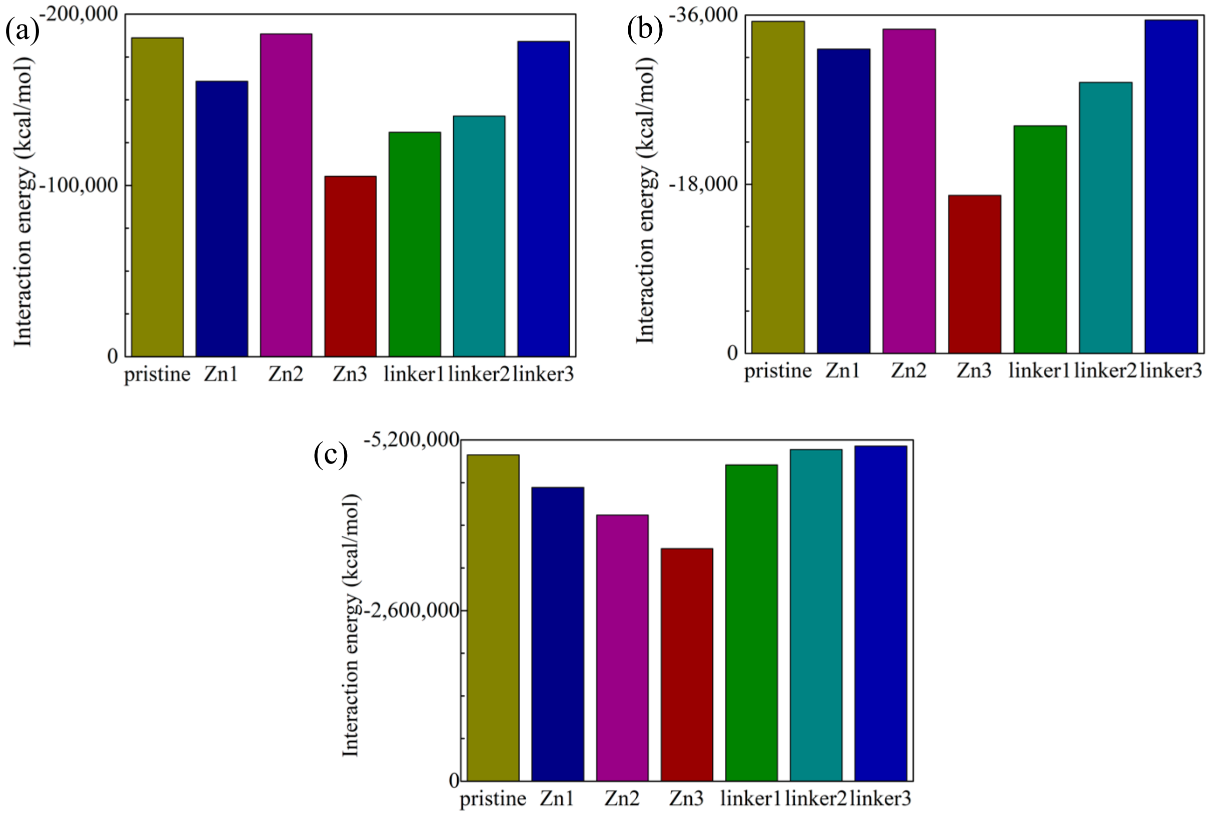

Figure S4. Interaction energy graphs of the respective ZIF-8s: (a) N atoms with the Na^+ ions, (b) Zn atoms with the Cl^- ions, and (c) Zn atoms with the O atoms of water molecules.

7. Interaction energy graphs between the ions and water molecules with the H3 atoms from the ZIF-8 membranes with Zn substitutional defects.

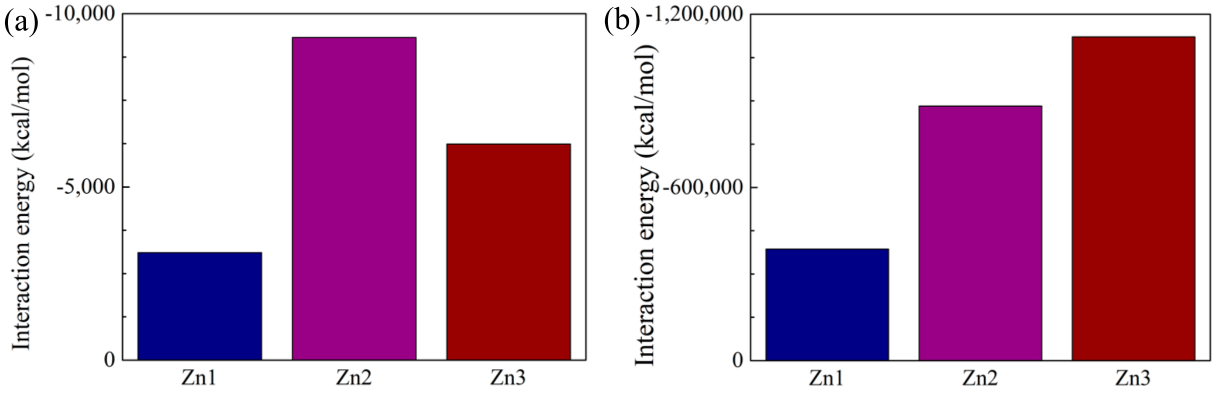

Figure S5. Interaction energy graphs of the respective ZIF-8s with number of Zn substitutional defect sites: (a) H3 atoms with the Cl^- ions and (b) H3 atoms with the O atoms of water molecules.

8. Interaction energy graphs between the ions and water molecules with the H3 atoms from the ZIF-8 membranes with linker substitutional defects.

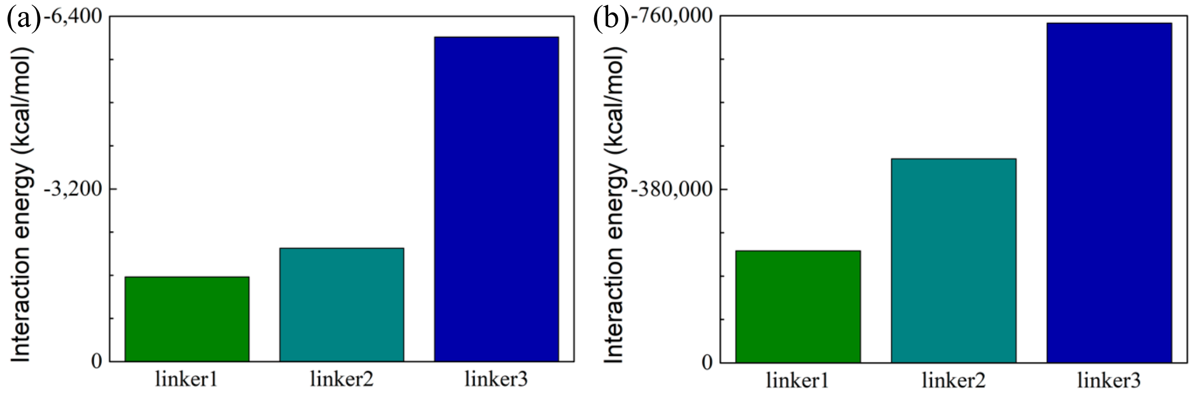

Figure S6. Interaction energy graphs of the respective ZIF-8s with number of linker substitutional defect sites: (a) H3 atoms with the Cl- ions and (b) H3 atoms with the O atoms of water molecules.

9. **Interaction energy graphs between the ions and water molecules with the H4 atoms from the ZIF-8 membranes with linker substitutional defects.**

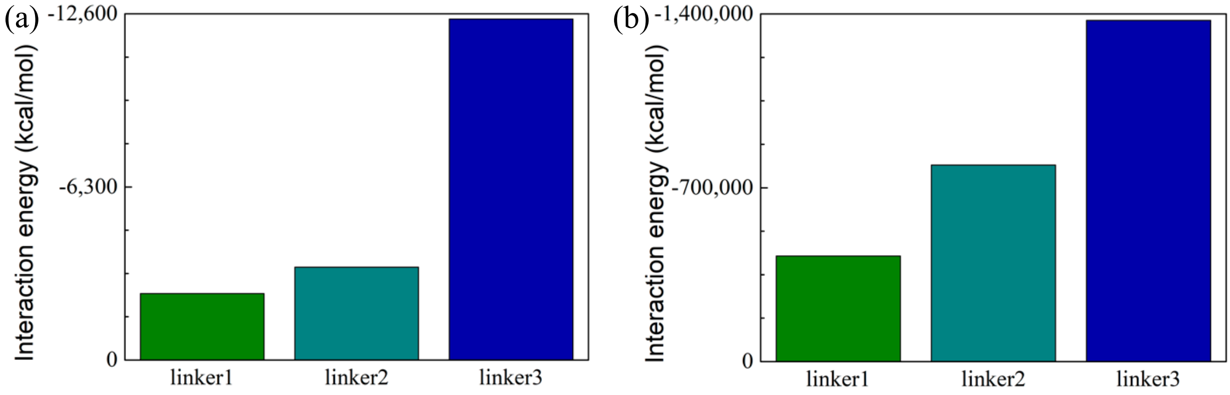

Figure S7. Interaction energy graphs of the respective ZIF-8s with number of linker substitutional defect sites: (a) H4 atoms with the Cl^- ions and (b) H4 atoms with the O atoms of water molecules.

10. **Interaction energy graphs between the ions and water molecules with the O2 atoms from the ZIF-8 membranes with linker substitutional defects.**

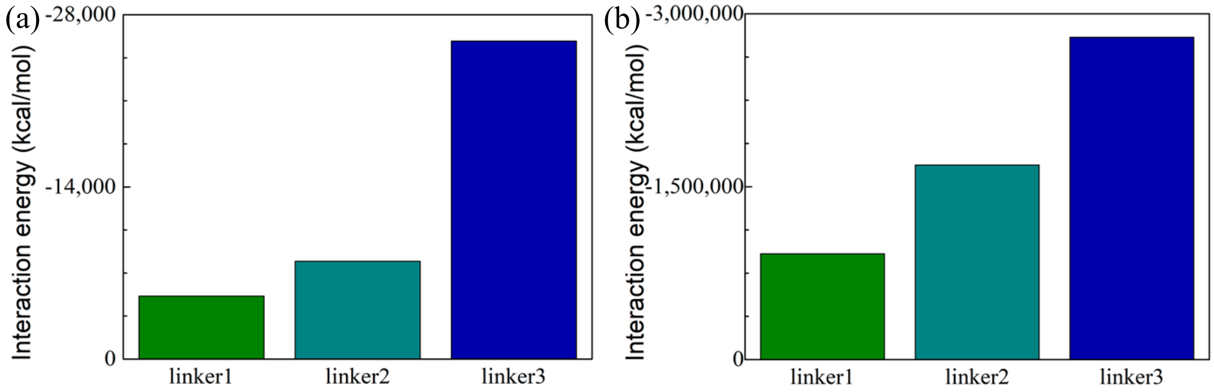

Figure S8. Interaction energy graphs of the respective ZIF-8s with number of linker substitutional defect sites: (a) O2 atoms with the Na^+ ions and (b) O2 atoms with the H atoms of water molecules.
